# Emotional intelligence and self-regulated learning: psychological pathways to student engagement and academic performance

**DOI:** 10.3389/fpsyg.2026.1917309

**Published:** 2026-08-21

**Authors:** Tao Yang, Qiaoyu Yang

**Affiliations:** 1 School of Educational Science, Quanzhou Normal University, Quanzhou, China; 2 Quanzhou Huayang Machinery Manufacturing Co., Ltd., Quanzhou, China

**Keywords:** academic burnout, academic engagement, academic performance, emotional intelligence, intervention, self-directed learning, self-efficacy, self-regulated learning

## Abstract

Emotional intelligence (EI) and self-regulated learning (SRL) have emerged as central constructs for explaining why some students sustain engagement and achieve academically under pressure while others succumb to burnout and inefficiency. This mini-review synthesizes theoretical and empirical work on the association between EI and SRL and on the psychological pathways through which the two jointly shape academic engagement and performance. We first situate the EI–SRL relationship within self-regulation theory and contrast the ability and trait models of EI, specifying how each maps onto distinct components of SRL—ability EI onto metacognitive monitoring and strategy selection, trait EI onto motivational persistence—and noting that evidence consistently supports a positive EI–SRL association but diverges on effect sizes, mediating mechanisms, and boundary conditions related to culture, educational level, and measurement. We then map the multiple mediating and moderating mechanisms, self-directed learning, academic and creative self-efficacy, the engagement–burnout balance, social support, and metacognitive regulation, that translate EI into academic outcomes, highlighting evidence that EI frequently exerts its influence indirectly and that the relative importance of each pathway varies with context and developmental stage. Because much of this evidence derives from nursing and medical samples, we also delimit the populations to which the pathways can currently be generalized. We further review intervention research indicating that EI and SRL are trainable competencies whose cultivation can improve performance and reduce burnout, while cautioning that methodological limitations may inflate reported effects. Finally, we identify key gaps, fragmented theory, neglect of cultural variation, scarcity of longitudinal designs, and the need for rigorous, technology-integrated interventions, and outline directions for moving from understanding psychological pathways to optimizing educational practice. Together, these findings position EI and SRL as core, cultivable psychological resources for student success and underscore the value of integrative, context-sensitive, and developmentally informed research.

## 1 Introduction

A long-standing question in educational psychology is why some students maintain sustained engagement and achieve strong performance when confronted with academic challenges, whereas others fall into burnout and inefficiency. Emotional intelligence (EI) and self-regulated learning (SRL) have increasingly attracted attention as key variables for explaining this variability. EI refers to the capacity to perceive, understand, manage, and use one's own and others' emotions, whereas SRL emphasizes the metacognitive ability of learners to proactively set goals, monitor the learning process, and adjust their strategies. Together, the two constitute important psychological resources that students draw on to cope with academic demands.

During the COVID-19 pandemic, the widespread adoption of remote learning exposed students to unprecedented emotional challenges and demands for autonomous study, further underscoring the critical role of EI and SRL in sustaining learning continuity (Albani et al., 2023; Pelikan et al., 2021). Nevertheless, the field remains divided over the direct association between EI and academic performance. Some studies confirm a positive correlation between EI and academic achievement (Alvi et al., 2023; Caballero-García and Ruiz, 2025), whereas others find that this direct effect is not significant and operates through mediating variables (Chang and Tsai, 2022). This controversy has prompted researchers to shift from a simple EI–performance dyad toward more complex analyses of psychological pathways. Variables such as SRL, learning motivation, and academic self-efficacy have been successively introduced to illuminate the mechanisms through which EI promotes academic success (Kim, 2022; Li et al., 2024; Shafait and Huang, 2022).

A further trend in current research is the conceptualization of EI and SRL as cultivable competencies rather than fixed traits. Intervention studies provide direct guidance for educational practice: systematically training students' emotion-management abilities and autonomous learning strategies can effectively improve academic performance and reduce burnout (Caballero-García and Ruiz, 2025; Molero Jurado et al., 2021; Powell et al., 2024).

This mini-review aims to synthesize the theoretical associations and empirical evidence linking EI and SRL, to analyze the psychological pathways through which the two jointly influence academic engagement and performance, to evaluate the effectiveness of existing interventions, and, on this basis, to identify research gaps and future directions.

## 2 Methods

### 2.1 Review design

This mini-review employed a narrative synthesis approach to integrate theoretical perspectives and empirical evidence on emotional intelligence, self-regulated learning, and academic outcomes. The review focused on identifying psychological pathways, contextual moderators, and intervention implications rather than estimating pooled effect sizes.

### 2.2 Literature search strategy

The literature search was conducted in PubMed, Web of Science, Scopus, and PsycINFO for English-language publications between January 2021 and February 2026. Search terms included emotional intelligence, self-regulated learning, self-directed learning, academic engagement, academic performance, academic achievement, academic burnout, self-efficacy, and intervention. The reference lists of retrieved articles were screened to identify additional relevant studies.

### 2.3 Study selection and narrative synthesis

Studies were eligible if they (i) were peer-reviewed empirical reports (cross-sectional, longitudinal, or intervention designs), systematic reviews, or meta-analyses; (ii) examined associations among EI, SRL, and academic engagement, performance, or burnout; and (iii) sampled secondary, undergraduate, or professional (e.g., nursing and medical) education populations. Records were excluded if they were conference abstracts, editorials, or non-peer-reviewed reports, if EI or SRL was not assessed as a distinct construct, or if the outcomes were unrelated to learning. Titles and abstracts were screened against these criteria, full texts of potentially eligible records were then assessed, and 28 sources were retained for the synthesis reported here; consistent with the mini-review format, no formal risk-of-bias appraisal was undertaken. The extracted information was synthesized narratively according to theoretical foundations, psychological mechanisms, intervention approaches, and contextual factors.

## 3 Results

### 3.1 Theoretical foundations of the EI–SRL association

The theoretical link between EI and SRL can be traced to a core assumption of self-regulation theory, which holds that individual behavior is driven not only by the external environment but also, and more fundamentally, by internal cognitive and emotional processes (Laulié et al., 2023). As the capacity to regulate one's own emotional states, EI helps learners maintain positive emotional experiences when facing academic challenges, thereby providing the affective foundation necessary for SRL.

Two principal theoretical models frame the EI–SRL relationship. The ability model conceptualizes EI as a set of measurable cognitive abilities comprising four dimensions: perceiving emotions, using emotions to facilitate thought, understanding emotions, and managing emotions. The trait model, by contrast, treats EI as a stable personality disposition, emphasizing individual differences in emotional experience and expression (Toriello et al., 2022). This distinction is consequential because the two models map onto different components of SRL. Ability EI, indexed by maximum-performance tests, captures how accurately learners appraise and reason about affective states, and should therefore align with the cognitive–metacognitive arm of SRL: recognizing that frustration signals a failing strategy, monitoring comprehension, and reallocating effort. Trait EI, indexed by self-report and best understood as emotional self-efficacy, captures confidence in one's emotional functioning, and should align with the motivational–volitional arm: task value and persistence under difficulty. The evidence is asymmetric on this point, since almost all studies reviewed below used trait or mixed self-report instruments; present conclusions therefore rest on perceived rather than demonstrated emotional competence, and the ability-model prediction remains largely untested.

Empirical research provides broad support for a positive EI–SRL association, although studies differ in effect sizes and specific mechanisms. In a study of 1,055 Italian university students, Albani et al. (2023) found that trait EI positively predicted all dimensions of SRL while negatively predicting perceived stress and intolerance of uncertainty. In 302 Korean nursing students, Kim (2022) further demonstrated a full mediation of SRL between EI and clinical performance.

The EI–SRL relationship is not, however, uniform across contexts. Studying faculty and staff at research universities in China and Pakistan, Shafait and colleagues found that the direct effect of EI on learning outcomes was non-significant, operating instead entirely through self-directed learning and knowledge-management processes (Shafait and Huang, 2022). This challenges the general assumption that EI directly promotes academic performance. In 240 health-sciences students, Ibrahim et al. (2024) showed that digital literacy, rather than self-regulation, mediated the relationship between EI and academic stress.

Part of the controversy surrounding the EI–performance relationship stems from measurement heterogeneity. Different EI scales (e.g., WLEIS, SREIT, TEIQue) assess different constructs, making findings difficult to compare directly (Toriello et al., 2022).

Gender differences represent another point of contention. Some studies report higher EI scores among women (Alvi et al., 2023; Sutil-Rodríguez et al., 2024), whereas others find that men score higher on certain emotion-management dimensions (Kurt and Eskimez, 2022); such discrepancies, like the inconsistent grade-level differences reported across studies (Kurt and Eskimez, 2022; Tang et al., 2025), may reflect cultural context and social expectations rather than essential differences in EI.

### 3.2 Psychological pathways linking EI, SRL, and academic outcomes

EI influences academic engagement and performance not through a single linear pathway but via multiple mediating and moderating mechanisms, including self-directed learning, academic and creative self-efficacy, the balance between academic engagement and burnout, social-support systems, and metacognitive regulation (Table 1).

**Table 1:** Psychological pathways linking emotional intelligence, self-regulated learning, and academic outcomes.

Pathway	Mechanistic role	Academic relevance	Predominant EI model in cited evidence	Representative evidence
Emotion regulation and SRL	EI helps students manage stress and uncertainty, sustaining goal setting, monitoring, attention control, and strategy adjustment.	Engagement, perceived competence, and clinical or academic performance.	Trait/self-report EI	Albani et al., 2023; Kim, 2022; Pelikan et al., 2021
Self-directed learning and metacognition	EI is translated into autonomous planning, learning responsibility, metacognitive awareness, and continuous strategy revision.	Learning outcomes, metacognitive awareness, lifelong-learning orientation.	Self-report (mixed-model) EI	Shafait and Huang, 2022; Li et al., 2024
Academic and creative self-efficacy	EI strengthens confidence, motivation, resilience, and creative problem-solving; effects may operate through chained mediation with SDL.	Academic achievement, psychological wellbeing, and clinical decision-making confidence.	Self-report EI (trait/mixed)	Chang and Tsai, 2022; Shengyao et al., 2024; Tang et al., 2025
Engagement–burnout balance and coping	Higher EI promotes resilient coping and positive engagement while reducing emotional exhaustion, frustration intolerance, and burnout risk.	Academic motivation, performance, and lower burnout.	Trait/perceived EI (self-report)	Molero Jurado et al., 2021; González-Yubero et al., 2025; Ruiz-Ortega and Berrios-Martos, 2025
Social support and relational engagement	EI facilitates interpersonal regulation and relationship building, increasing access to peer, family, and teacher support.	Cognitive outcomes, lower academic stress, and stronger relational engagement.	Self-report EI	Iqbal et al., 2021; Ullah et al., 2023
Boundary conditions and intervention implications	Culture, educational level, measurement tools, digital literacy, and metacognitive self-regulation may alter pathway strength and intervention response.	Explains heterogeneity in EI–performance links and supports targeted EI/SRL training.	Both ability and trait measures represented (review-level)	Toriello et al., 2022; Ibrahim et al., 2024; Ko, 2026; Mehler et al., 2024; Powell et al., 2024

EI, emotional intelligence; SRL, self-regulated learning; SDL, self-directed learning. The table summarizes mechanisms discussed in Section 3 and is not intended as a quantitative ranking of pathway strength.

#### 3.2.1 Self-directed learning

The mediating role of self-directed learning is among the best-established pathways. In research on faculty at Chinese research universities, Shafait and Huang (2022) found that EI indirectly influenced learning outcomes through learning orientation (i.e., commitment to learning), with self-directed learning fully mediating the EI–outcome relationship. In 600 Chinese nursing students, Li et al. (2024) confirmed that self-directed learning ability mediated the relationships among educational environment, learning motivation, EI, and metacognitive awareness.

#### 3.2.2 Academic and creative self-efficacy

Academic and creative self-efficacy constitute a second important mediating pathway. In 518 Chinese university students, Shengyao et al. (2024) found that self-efficacy, motivation, and resilience mediated the relationships between EI and both psychological wellbeing and academic achievement. In 1,126 nurses, Tang et al. (2025) revealed a chain-mediation effect of creative self-efficacy between EI and clinical decision-making confidence: EI enhanced creative self-efficacy, which in turn strengthened self-directed learning and ultimately promoted clinical decision-making confidence.

#### 3.2.3 The engagement–burnout balance

The balance between academic engagement and burnout offers another important lens. Drawing on the job demands–resources model, Ruiz-Ortega and Berrios-Martos (2025) used structural equation modeling with 630 Spanish university students and found that EI both directly and positively predicted academic performance and operated indirectly by increasing engagement and reducing burnout; conversely, frustration intolerance negatively affected performance by increasing burnout and reducing engagement. González-Yubero et al. (2025) further found that resilient coping partially mediated the relationship between EI and academic motivation, with higher-EI students more likely to adopt effective coping strategies and thereby sustain intrinsic motivation.

#### 3.2.4 Social support and relational engagement

Social support and relational engagement constitute a sociopsychological pathway through which EI affects academic outcomes. In 338 Chinese undergraduates, Iqbal et al. (2021) found that relational engagement played a key mediating role between EI and cognitive outcomes, as higher-EI students were able to build better peer and student–teacher relationships and thus obtain more learning support. In 429 Bangladeshi students, Ullah et al. (2023) revealed a mediating effect of social support between EI and academic stress.

#### 3.2.5 Moderating mechanisms

The presence of moderating mechanisms further enriches the analysis of these mediating pathways. In 432 undergraduates, Ko (2026) found that metacognitive self-regulation moderated the relationship between digital learning competence and informal digital learning engagement, with students high in metacognitive self-regulation more effectively translating digital competence into autonomous learning behavior. Laulié et al. (2023) found that EI moderated the relationship between psychological-contract fulfillment and emotional exhaustion, such that only individuals high in EI benefited from contract fulfillment.

Notably, the relative importance of these pathways varies by context: in high-stress clinical settings the mediating role of creative self-efficacy is stronger, whereas in lower-stress settings self-directed learning is more prominent, and the chain-mediation effect is most pronounced among less experienced nurses, with the direct effect of EI strengthening as experience accumulates (Tang et al., 2025). The pathways of EI thus appear context-dependent and developmentally specific.

### 3.3 Intervention evidence

Researchers have developed and evaluated a range of intervention programs aimed at enhancing EI and SRL.

#### 3.3.1 Curriculum-integrated EI training

Curriculum-integrated EI training is the most common form of intervention. In a systematic review and meta-analysis of 50 workplace emotional-competence training studies, Mehler et al. (2024) found a moderate post-intervention improvement in EI (SMD = 0.44) that persisted at 3-month follow-up. Effects did not differ significantly across programs targeting EI, empathy, or emotion regulation. A meta-analysis by Powell et al. (2024) of healthcare workers likewise reported positive intervention effects but noted that methodological limitations may lead to overestimation.

#### 3.3.2 EI development through physical education

A systematic review by Rico-González (2023) analyzed 28 studies of EI in physical education and identified four effective instructional models: pedagogy based on bodily expression and relaxation, cooperative-learning models (e.g., the Sport Education Model), community-engagement interventions (e.g., social-emotional learning and the Teaching Personal and Social Responsibility model), and augmented-reality games. A shared feature of these models is their emphasis on collaboration, care for others, and social wellbeing, indicating that EI development requires authentic social-interaction contexts.

#### 3.3.3 Interventions for specific student populations

Interventions targeting specific student populations have also yielded positive outcomes. Implementing a positive-emotion intervention with 300 Spanish university students, Caballero-García and Ruiz (2025) found that the experimental group scored significantly higher than controls on EI and wellbeing. A randomized controlled trial by Molero Jurado et al. (2021) with 1,287 high-school students showed that stress management and emotional states within EI mediated the relationship between academic performance and burnout, suggesting that emotion-management interventions for academically struggling students may help prevent burnout.

#### 3.3.4 Strategies to promote self-regulated learning

Strategies to promote SRL have likewise attracted attention. A systematic review by Wong et al. (2021) identified factors associated with self-directed learning in undergraduate nursing students, spanning educational-environment factors (curriculum type, study length, teaching strategies) and personal factors (age, problem-solving ability, self-efficacy, and learning attitudes). Effective intervention strategies included autonomous learning, group learning, and ongoing educator support. Drawing on experience with India's competency-based medical curriculum, Charokar and Dulloo (2022) emphasized the importance of explicitly embedding self-directed learning in curriculum design, supported by faculty development, resource provision, and aligned cognitive and affective assessment.

#### 3.3.5 Conditionality of intervention effects

The conditionality of intervention effects is an important finding of current research. Not all students benefit equally from interventions; individuals' baseline levels, learning environments, and intervention dosage all influence outcomes. Iacolino et al. (2023) found that metacognitive ability moderated the relationship between remote-working risk factors and EI, with teachers high in metacognitive ability better able to maintain their EI levels under the pressures of remote teaching.

Despite this progress, several methodological limitations remain. Most studies employ quasi-experimental rather than randomized controlled designs, have limited sample sizes, and lack long-term follow-up data (Mehler et al., 2024; Powell et al., 2024). Moreover, the measurement of intervention effects relies largely on self-report scales, leaving findings susceptible to social-desirability effects.

## 4 Discussion

The synthesized findings indicate that emotional intelligence and self-regulated learning constitute interconnected psychological resources supporting academic engagement and performance. Emotional intelligence appears to influence academic outcomes through multiple pathways involving emotion regulation, metacognition, self-efficacy, motivation, and social processes.

### 4.1 Theoretical and educational implications

The evidence extends educational psychology models by emphasizing the transition from emotional resources to learning behaviors and academic outcomes. Integrating emotional intelligence development with self-regulated learning strategies may provide a more comprehensive approach for student development.

Three practices follow directly for everyday university teaching. First, pair strategy instruction with an emotional debrief: when planning, monitoring, and revision strategies are taught, ask students to name the emotion that typically derails each stage and to record one regulation move beside each strategy. Second, embed brief affective check-ins in existing formative assessment—two items on anticipated difficulty and current confidence attached to a weekly quiz—and return them with feedback, so that appraisal and reappraisal become routine rather than remedial. Third, timetable short emotion-management sessions at the transition points where burnout risk peaks, such as the first clinical placement or the first major examination, delivered by course teachers rather than offered as an optional co-curricular extra. Each can be accommodated within existing contact hours.

### 4.2 Sample heterogeneity and generalizability of the evidence

A substantial share of the evidence reviewed here comes from nursing, medical, and other health-professions samples (Kim, 2022; Wong et al., 2021; Li et al., 2024; Sutil-Rodríguez et al., 2024; Tang et al., 2025). These populations are not representative of higher education generally: clinical training combines high-stakes patient contact, emotionally demanding placements, structured competency assessment, and early professional-identity formation, conditions that plausibly raise the emotional load on self-regulation and may therefore strengthen the observed EI–SRL–outcome couplings. Two further boundaries deserve note: Shafait and Huang (2022) sampled university faculty and staff, and Tang et al. (2025) practicing nurses, so these pathways describe adult professional learners rather than students.

Consistent with this, studies of general undergraduate populations report the same direction of effect but a more prominent direct EI–performance path (Shengyao et al., 2024; Ruiz-Ortega and Berrios-Martos, 2025), whereas full mediation through SRL is reported most consistently in clinical and professional samples (Kim, 2022; Shafait and Huang, 2022). The present findings should therefore not be transferred uncritically to K−12 settings, where SRL is still consolidating and adult supervision remains substantial, or to humanities and STEM undergraduates, whose assessment regimes are less emotionally loaded. Cross-disciplinary comparisons within a single design are needed before these pathways can be treated as general.

### 4.3 Limitations and future directions

A major limitation of current research is insufficient theoretical integration. EI research has long been divided between ability and trait models, whereas SRL research is dispersed across cognitive, metacognitive, and motivational domains. This fragmentation makes findings difficult to integrate and limits the systematicity of intervention design. The dimension-level correspondences outlined in Section 3.1 need to be tested directly, ideally by administering performance-based and self-report EI measures in the same sample alongside separate indices of metacognitive and motivational regulation.

A second important gap is the neglect of cultural variation. Existing research concentrates mainly on Western or East Asian contexts and lacks systematic cross-cultural evidence. The comparison by Shafait and Huang (2022) of research universities in China and Pakistan revealed country-level differences in the EI–outcome relationship, suggesting that cultural values, educational traditions, and social norms may moderate the pathways of EI.

The scarcity of longitudinal mechanistic research limits firm causal inference. A cross-lagged analysis by Cheng et al. (2024) of 177 gifted children showed that trait EI positively predicted subsequent creative self-efficacy rather than the reverse, yet comparable studies in general student populations remain scarce. Multi-wave longitudinal designs are needed to track the developmental trajectories of EI and SRL and to inform the optimal timing of interventions.

Deepening intervention research is a further priority. Although meta-analyses confirm the effectiveness of EI training, most studies suffer from methodological limitations, including the absence of randomized control, small samples, and short follow-up (Mehler et al., 2024; Powell et al., 2024). Future research should prioritize rigorously designed randomized controlled trials, incorporate active control groups to account for nonspecific effects, and extend follow-up periods to assess the durability of effects. Outcome assessment should likewise move beyond self-report to include behavioral observation and academic-performance records. Greater precision in intervention content—clarifying whether training targets emotional perception, understanding, or management—would help isolate the active ingredients.

The potential of technology integration remains underexploited. Digital learning environments offer new tools for promoting EI and SRL. Future research could explore how to embed EI training within digital platforms, using real-time emotional feedback and personalized learning paths to enhance intervention effects.

Finally, the necessity of interdisciplinary collaboration is increasingly apparent. Research on the neural basis of EI and SRL—such as the interaction between the prefrontal cortex and the limbic system—offers a biological account of their association (González-Yubero et al., 2025). Future work should integrate perspectives from education, psychology, cognitive neuroscience, and computer science to develop more explanatory models and more effective interventions.

## 5 Conclusion

In sum, EI and SRL constitute core psychological pathways for promoting students' academic success. By clarifying conceptual boundaries, developing more context-sensitive theoretical models, and designing more rigorous and individualized interventions, future research can ultimately advance the translation from understanding psychological pathways to optimizing educational practice.
